# Seroprevalence and risk factors of *Neospora caninum* infection among domestic sheep in Henan province, central China

**DOI:** 10.1051/parasite/2018019

**Published:** 2018-03-20

**Authors:** Shuai Wang, Lingjuan Li, Yi Lu, Haizhu Zhang, Qing Xie, Zhenchao Zhang

**Affiliations:** 1 School of Basic Medical Sciences, Xinxiang Medical University, Xinxiang, Henan, 453003 PR China; 2 Henan Muxiang Veterinary Pharmaceutical Co., Ltd, Zhengzhou, Henan, 450000 PR China; 3 Animal Disease Prevention and Control Center of Xinxiang City, Xinxiang, Henan, 453003 PR China

**Keywords:** *Neospora caninum*, seroprevalence, sheep, ELISA, central China

## Abstract

This study aimed to determine the frequency of antibodies to *Neospora caninum* in domestic sheep raised in Henan province, central China. Serum samples from 779 domestic sheep were collected from March 2015 to May 2016, and antibodies to *N. caninum* were evaluated using an enzyme-linked immunosorbent assay (ELISA). The results showed an overall IgG positive rate of 7.32% (57/779). The risk factors significantly related to seropositivity to *N. caninum* in sheep were the age, the presence of dogs, and the rearing system. This is the first report of *N. caninum* infection and associated risk factors in domestic sheep in central China.

## Abstract (fr)

Cette étude visait à déterminer la fréquence des anticorps contre *Neospora caninum* chez les moutons domestiques élevés dans la province du Henan, en Chine centrale. Des échantillons de sérum provenant de 779 moutons domestiques ont été prélevés entre mars 2015 et mai 2016, et les anticorps dirigés contre *N. caninum* ont été évalués à l’aide du test ELISA. Les résultats ont montré un taux global d’IgG positif de 7,32% (57/779). Les facteurs de risque significativement liés à la séropositivité à *N. caninum* chez les moutons étaient l’âge, la présence de chiens et le système d’élevage. Ceci est le premier rapport sur l’infection par *N. caninum* et les facteurs de risque associés chez les moutons domestiques en Chine centrale.

## Introduction

*Neospora caninum* is an obligate intracellular protozoan parasite, which is regarded as one of the leading infectious causes of abortion among cattle around the world [10,22]. It has been detected in a variety of domestic and wild or free living animals, including cattle, goats, sheep, horses and deer [5,7,15,21].

Although cattle represent the most relevant and economically important target host for *N. caninum* [22,29], the presence of *N. caninum* infection and transplacental transmission in sheep have also been reported [6,26]. *N. caninum* was first described as a natural infection in sheep in a congenitally infected lamb in England [8]. Soon afterwards, neosporosis of natural occurrence among sheep was discovered around the world [4,14]. Like the parasite *Toxoplasma gondii*, *N. caninum* was also associated with reproductive failure. Traditionally, *N. caninum* is considered the main cause of abortion and neonatal death in cattle, whereas *T. gondii* has been thought to be one of the principal agents causing abortion in sheep. However, it was also suggested by recent evidence that *N. caninum* played an essential role in miscarriage among sheep [11,12].

Although the seroprevalence of *N. caninum* in sheep has been reported worldwide [1,17,19], limited information is available on the seroprevalence of *N. caninum* in sheep in China. So far, only the seroprevalence of *N. caninum* in sheep raised in Qinghai province, western China has been investigated. In total, 10.33% (62/600) of the sheep from Qinghai province were seropositive for *N caninum.* [13]. Data on the seroprevalence of *N. caninum* among sheep raised in other provinces of China remain unknown.

Therefore, this study was conducted to investigate the seroprevalence and the risk factors related to seropositivity of *N. caninum* among domestic sheep in Henan province, central China. The results will lay the groundwork for controlling *N. caninum* infections among domestic sheep in this region.

## Materials and methods

### Ethics statement

The Ethics Committee of Xinxiang Medical University has reviewed and approved this study (reference no. 2015018).

### The location of this study

**Figure 1:**
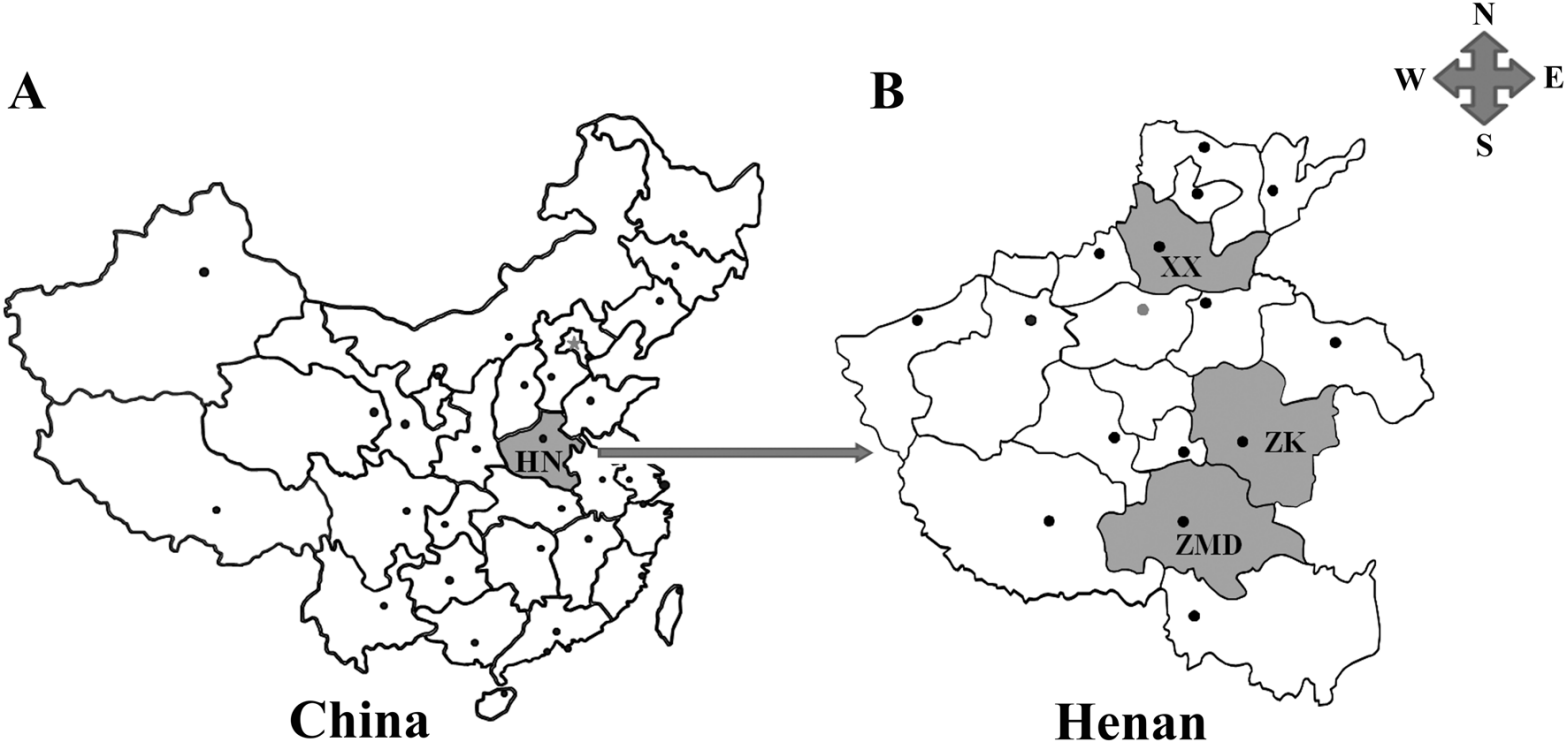
Geographic distribution of the sampling sites in Henan province, China used in this study. A: Henan province (HN, shadowed areas) is located in the central part of mainland China. B: Shadowed areas are the sampling locations for the present survey. XX: Xinxiang; ZK: Zhoukou; ZMD: Zhumadian.

The current investigation was performed in Henan province which is situated in the central section of China (Fig. 1), with northern latitude of 31°23′−36°22′ and eastern longitude of 110°21′−116°39′. It occupies an area of 167,000 km^2^ and has a population of about 106.01 million with the Yellow River passing through its central section. The continental monsoon climate is characterized by two distinct seasons, with average precipitation of 530–1380 mm and annual temperatures of 12.1–15.7 °C. There are seventeen provincial cities distributed in Henan province, with the city of Zhengzhou as its capital. Three cities including Xinxiang (35°18′N, 113°54′E), Zhoukou (33°03′−34°20′N, 114°05′−115°39′E) and Zhumadian (32°18′−33°35′N, 113°10′−115°12′E) were chosen for the collection of specimens since these cities are the main suppliers of ovine meat to Henan province and the neighboring regions.

### Sample collection

In total, 779 blood samples were collected from domestic sheep in Henan in the above-mentioned cities, throughout the period from March 2015 to May 2016. To identify the risk factors, the information on sources, gender, age, rearing system of each animal, as well as the presence of dogs in the herd was also collected. After centrifuging blood specimens, serum was collected and preserved in 1.5 mL Eppendorf tubes at a temperature of −80°C, before being tested for antibodies against *N. caninum*.

### Detection of *N. caninum* antibodies

The commercial ELISA kit (*Neospora* Ab Test, IDEXX Laboratories Inc., Westbrook, ME, USA) was used to analyze serum samples (diluted 1:100 in sample buffer), based on the manufacturer’s instructions. The IDEXX *Neospora* Ab Test is an enzyme immunoassay for the detection of antibodies against *N. caninum* in serum and plasma samples of ruminants (cattle, goats, and sheep). The rate of specimen absorbance to positive control absorbance (S/P ratio) was represented as the result. S/P = OD_sample_ − OD_negative control_/OD_positive control_ − OD_negative control_. The S/P ratio of 0.50 was considered the threshold value of *N. caninum* infection and any specimen with a ratio higher than that was considered positive. The specificity and sensitivity of this *N. caninum* ELISA kit were 98.3% and 98.6%, respectively [2].

### Statistical analyses

A Chi square test was used to analyze variations of effects on *N. caninum* serum positive rates by risk factors including source, age, gender, rearing system of each animal, and the presence of dogs in the herd. Statistical analyses were performed using the SPSS 20 software for Windows (SPSS Inc., Chicago, IL, USA). A *p* value lower than 0.05 indicated statistical significance.

## Results

In this study, ELISA was conducted on 779 blood samples from domestic sheep to test for *N. caninum* antibodies. As shown in Table 1, the serum positive rate of *N. caninum* among domestic sheep from Henan province, central China was 7.32% (57/779). Seropositive rates for sheep from the cities of Zhoukou, Zhumadian, and Xinxiang were 4.71% of 255, 8.82% of 272, and 8.33% of 252, respectively.

**Table 1:** Seroprevalence of *Neospora caninum* in 779 domestic sheep in Henan province, central China.

Variable	No. examined	No. positive	Prevalence (%)	X^2^	*P*-value
Region					
Zhoukou	255	12	4.71	3.858	0.145
Zhumadian	272	24	8.82		
Xinxiang	252	21	8.33		
Gender					
Male	292	18	6.16	0.915	0.339
Female	487	39	8.01		
Age (years)					
≤ 1	246	11	4.47	8.587	0.014
1∼2	292	19	6.51		
≥ 2	241	27	11.2		
Presence of dogs in the herd					
Yes	359	40	11.14	14.365	< 0.001
No	420	17	4.05		
Rearing system					
Extensive	215	25	11.63	8.845	0.012
Semi-intensive	343	22	6.41		
Intensive	221	10	4.52		
Total	779	57	7.32		

The serum positive rate for *N. caninum* among female sheep (8.01%, 39/487) was higher than that among male sheep (6.16%, 18/292) (Table 1), but the difference was not statistically significant (*p*>0.05). The serum positive rate of *N. caninum* among sheep rose remarkably (*p* < 0.05) with increasing age. The peak value (11.20%) appeared among sheep no younger than 2 years, followed closely by the intermediate value (6.51%) which appeared among 1–2 year-old sheep. The sheep younger than 1 year exhibited the lowest prevalence value, at 4.47% (Table 1).

In addition, significantly higher seroprevalence was found in sheep raised on the farm with the presence of dogs (11.14%), compared to those without dogs (4.05%) (*p* < 0.01). Additionally, the seroprevalence obtained in extensively raised sheep was higher than that in intensively and semi-intensively raised samples (*p* < 0.05), but the difference between intensively and semi-intensively raised sheep was not significant (*p *> 0.05) (Table 1).

## Discussion

The current investigation showed that the total serum positive rate of *N. caninum* was 7.32% among domestic sheep in Henan, central China, which was lower than that found in Qinghai province, China (10.33%) [13]. Compared with other countries in the world, it was lower than rates observed in Galicia, Northwest Spain (10.1%) [20], the Czech Republic (12%) [3], São Paulo, Brazil (59.23%) [19], Pernambuco State, Brazil (64.2%) [27], but higher than those observed in New South Wales, Australia (2.2%) [4] and Slovakia (3.7%) [25]. The variations in serum positive rates among different regions are probably related to different sheep breeds, sample capacities, times of investigations, testing methods, as well as geographical and ecological factors.

Prevalence of *N. caninum* in this work was not found to be associated with gender of sheep, which was consistent with previous results [23,24]. Moreover, the *N. caninum* serum positive rate among sheep in this study was remarkably correlated with increasing age in a positive manner, which was in line with results in previous reports [19,24,27], indicating that *N. caninum* was probably transmitted horizontally among the investigated herds.

Dogs play an essential role in *N. caninum* transmission since they are the final hosts and shed oocysts into the environment [14,18,28]. Sheep can be infected with *N. caninum* mainly by intake of drink and food containing sporulated oocysts of *N. caninum,* or by transplacental transmission [11,14,16]. In this study, canine presence on sheep farms was confirmed as one of the risk factors for occurrence of *N. caninum* infection among sheep, which agreed with findings in previous studies [1,14,24].

Moreover, the serum positive rate among extensively raised sheep was higher than that among intensively and semi-intensively raised samples (*p* < 0.05), which was consistent with results in previous reports [9,19]. These results suggest that the rearing system is certainly a very important risk factor associated with *N. caninum* infection in sheep. Intensive sheep farms in central China may have a high level of hygiene, preventing oocysts of *N. caninum* from being transmitting among herds. On the other hand, semi-intensive sheep farms are usually family-run businesses, which probably have lower hygiene standards and consequently suffer from *N. caninum* oocysts spreading among their animals. Extensive sheep farms may be more exposed to dogs in the environment or to contaminated stagnant pools, even though oocysts may be more dispersed in the environment.

## Conclusions

The existence of *N. caninum* among sheep in Henan province, central China was revealed for the first time in the current study. Control measures, for example, completely cutting off contact between canines and sheep, are needed on sheep farms. Furthermore, further studies will be needed to determine the impact of *N. caninum* on sheep reproduction disorders in China.

## Conflict of interest statement

The authors declare that they have no conflicts of interest in relation to this article.
